# Philosophical bias is the one bias that science cannot avoid

**DOI:** 10.7554/eLife.44929

**Published:** 2019-03-13

**Authors:** Fredrik Andersen, Rani Lill Anjum, Elena Rocca

**Affiliations:** 1 NMBU Centre for Applied Philosophy of Science, School of Economics and BusinessNorwegian University of Life SciencesAasNorway; 2 Faculty of Health and WelfareØstfold University CollegeHaldenNorway

**Keywords:** philosophy of science, philosophy of biology, bias

## Abstract

Scientists seek to eliminate all forms of bias from their research. However, all scientists also make assumptions of a non-empirical nature about topics such as causality, determinism and reductionism when conducting research. Here, we argue that since these 'philosophical biases' cannot be avoided, they need to be debated critically by scientists and philosophers of science.

Scientists are keen to avoid bias of any kind because they threaten scientific ideals such as objectivity, transparency and rationality. The scientific community has made substantial efforts to detect, explicate and critically examine different types of biases (Sackett, 1979; Ioannidis, 2005; Ioannidis, 2018; Macleod et al., 2015). One example of this is the catalogue of all the biases that affect medical evidence compiled by the Centre for Evidence Based Medicine at Oxford University (catalogueofbias.org). Such awareness is commonly seen as a crucial step towards making science objective, transparent and free from bias.

There is, however, one exception to this rule, which we call 'philosophical bias'. These are *b*asic *i*mplicit *a*ssumptions in *s*cience about how the world is (ontology), what we can know about it (epistemology), or how science ought to be practiced (norms). As we shall see, philosophical biases influence, justify and enable scientific practice: in short, they are an integral part of science.

Basic philosophical assumptions count as biases because they skew the development of hypotheses, the design of experiments, the evaluation of evidence, and the interpretation of results in specific directions. In our own research, we look at biases related to ontological, epistemological and normative assumptions about causality, probability and complexity. To give an example related to causality: when choosing a scientific method to establish a causal relationship between some medical condition and a virus, one must first have an idea of what causality is. This is a part of science that cannot be discovered empirically, but remains tacitly assumed in scientific methodology and practice.

## Examples of philosophical bias

Doing science without making any basic philosophical assumptions is impossible. But are all philosophical assumptions biases? No. Sometimes these assumptions are chosen deliberately and explicitly by the scientist, and used as auxiliary premises for theoretical purposes. For instance, one might adopt a philosophical assumption such as determinism to make a certain model work. Determinism is the assumption that, given a set of initial conditions, there is only one possible outcome. For instance, we could build a model of population growth which assumes that growth is completely determined by the initial population density: any deviations from the predictions of this model could, therefore, be taken as evidence that factors other than the initial conditions have an influence on population growth (Higgins et al., 1997). So even if one does not believe that determinism or some other philosophical assumption is true in all situations, making such an assumption can still serve a purpose.

When philosophical premises are chosen explicitly and purposely in this way, we would not call them 'biases'. In most cases, however, scientists remain unaware of these assumptions and of how they influence research. When a philosophical premise is implicitly accepted in our theories and methods, it becomes a philosophical bias. How does this affect the life sciences?

Philosophical biases are typically acquired from science education, professional practice or other disciplinary traditions that define a scientific paradigm. This is why scientists with varying backgrounds might adopt different philosophical biases. Biology, for example, is concerned with both entities and processes (Nicholson and Dupré, 2018). The standard ontological assumption is that entities (such as proteins) are more fundamental than processes, and that processes are produced by interacting entities. Molecular biologists have traditionally taken this as the default position. The ability of entities, such as proteins, to interact with each other is determined by their chemical structure, so to understand processes (such as the interactions between proteins), we need to understand the entities themselves in detail.

Doing science without making any basic philosophical assumptions is impossible. But are all philosophical assumptions biases?

However, some scientists take the view that processes are more fundamental than entities (Guttinger, 2018). In this view, entities are understood as being the result of processes that are stable over some length of time, and the best way to understand the behavior of an entity is to study the relations it has with other entities, rather than its internal structure. Ecologists tend to take this view, thinking in terms of systems in which the properties of individuals and species are determined by their relationships with each other and their environment.

The tension between these two ontological positions is not a purely philosophical or abstract point, it can have practical consequences. Ecologists and molecular biologists, for example, had different views about GM crops in early debates about their safety: ecologists focused on the unpredictability of environmental effects caused by GM crops, and had no strong opinions on similarities and differences between GM crops and conventional crops. Molecular biologists, on the other hand, stressed the fundamental equivalence between GM crops and conventional crops, while dismissing issues related to the predictability of environmental effects (Kvakkestad et al., 2007). Two of the present authors (ER and FA) have studied a similar clash of philosophical biases in the debate about the safety of stacked GM plants (that is, plants where conventional breeding techniques are applied to GM plants; Rocca and Andersen, 2017). One school of thought viewed the new plant as a conventional hybrid and argued that, in most cases, one can deduce the safety of the new plant from knowledge of the safety of its parental GM plants. This means thinking about complexity as being various combinations of unchanging parts. The other school, however, argued that one cannot deduce the safety of the new plant from the safety of the parental GM plants. Here, complexity is thought of as an emergent matter where parts lose their properties and identity in the process of interaction.

It is crucial that decision-makers (such as governments and regulatory agencies) are aware of these non-empirical aspects of science when introducing laws and regulations in controversial areas.

## Philosophical debates in science and medicine

Do scientists usually care about philosophical biases? In *The Structure of Scientific Revolutions* Thomas Kuhn introduced the idea of paradigms and paradigm shifts in science. Within a scientific paradigm, there is general consensus among researchers about the main theories, central concepts, relevant research questions, standard research procedures and basic mechanisms. Kuhn called this phase 'normal science', and argued that the role of the scientist was to fill in the gaps in our knowledge within the paradigm. Therefore, in times of normal science, there is little need for or interest in philosophical discussions on the foundations of a subject. However, according to Kuhn, when scientists start engaging in philosophical debates about their subject, a paradigm shift might be imminent (Kuhn, 1962). The most famous example of a paradigm shift is probably the emergence of quantum theory in physics, which challenged basic assumptions concerning the nature of causation, time, space and determinism. Philosophical debates between Einstein, Bohr and others had a central role in the development of quantum theory.

Normally, awareness is the first step towards overcoming some form of bias. However, this does not work in the case of philosophical biases.

Ongoing philosophical debates in medicine about a number of topics — such as approaches for informing medical decisions, models for understanding health and illness, and scientific norms for gathering medical knowledge (Greenhalgh et al., 2014; Loughlin et al., 2018; Anjum and Mumford, 2018) — might indicate that there is a paradigmatic crisis. One discussion is about the biomedical model of health and illness, which has been a dominant view in medicine for many decades. Critics of this model have argued that it is reductionist in nature, meaning that medical causes and medically relevant explanations are limited to the physical level, thus ignoring the causal influence of psychological, social and other higher-level factors on human biology (Engel, 1977).

Another philosophical debate in medicine concerns randomized controlled trials (RCTs) and their assumed status as the gold standard for establishing causation. In an RCT, an intervention is understood to be causal if the outcomes for the test group (the group receiving the intervention) and the outcomes for the control group are different in a statistically significant way. According to the norms of evidence-based medicine, the results from RCTs should guide clinical decisions about individual patients (Howick, 2011). However, this immediately leads to a tension between the public health perspective, where health advice is given at the level of populations, and the clinical perspective, where health advice is given to individual patients. It could be argued that treating a patient on the basis of what works best for a group is an example of the ecological fallacy; that is, of inferring from group to individual. However, this inference is valid under a philosophical assumption called frequentism, according to which individual propensities are derived from statistical frequencies. In this way, tensions in medical thinking and practice can have their origins in ontological, epistemological and normative biases.

## Should science aim to overcome philosophical biases?

Normally, awareness is the first step towards overcoming some form of bias. However, this does not work in the case of philosophical biases. We saw that basic assumptions are fundamental premises for science. They represent the lens through which we see new information. So even when these assumptions are explicated and challenged, all we can do is replace them with alternative biases. In denying dualism, reductionism or determinism, for instance, one still has to adopt an alternative, such as holism, emergence or indeterminism. Why should scientists inconvenience themselves with this process?

First, explicating philosophical biases is useful because it reveals competing perspectives (Douglas, 2000). This is crucial for scientific progress. Moreover, it also stops science from becoming a dogmatic enterprise. For instance, the health hazard from exposure to complex chemical mixtures, such as petroleum, has traditionally been calculated by grouping its components in fractions with similar chemical properties and, therefore, similar intrinsic toxicity and bioavailability. Each fraction is assigned a 'reference dose', which is the maximum dose considered safe (based on laboratory experiments and short-term monitoring after previous oil spills), and a mathematical formula is then used to combine the reference doses for each fraction and predict the health hazard from the mixture (Vorhees and Butler, 1999).

However, this approach to calculating health hazards is basically a form of reductionism (Hohwy and Kallestrup, 2008) because it is based on breaking down a chemical mixture into smaller parts, analyzing these parts in isolation, and then recombining them. This approach was considered for a long time the most scientifically reliable, but more recently the assumption that the most reductionist methodologies are also the most scientifically reliable has been questioned (Peterson et al., 2003). A competing assumption is that new hazards emerge at the level of the whole that cannot be found by studying its parts: this means that interactions between the parts can lead to changes within the parts themselves, and also to changes of the whole (Anjum and Mumford, 2017). This latter approach, based on philosophical assumptions different to those that underpin the traditional approach, led to a form of hazard prediction called ecosystem-based toxicology (Peterson et al., 2003).

Second, philosophical biases can influence the evaluation of scientific results, especially when the biases are epistemological in nature. Given the same evidence, some scientists might consider reliability, or internal validity, as the most important epistemic quality. Generally, these scientists require evidence from RCTs, where confounding factors are strictly controlled, in order to claim causation, and they tend to be skeptical about epidemiological evidence (Allmers et al., 2009). Other scientists might prefer to have converging evidence from more than one type of method, such as a combination of epidemiological evidence, a dose-response relationship and a plausible mechanisms (Osimani and Mignini, 2015). And still, other scientists might emphasize external validity, with evidence from a representative sample of relevant cases, plus evidence of a causal mechanism, being sufficient to establish causation (Anjum and Rocca, 2018; Hicks, 2015; Edwards, 2018). Scientists supporting any one of these stances should ideally be able to argue for why their epistemological bias should be considered superior. Awareness of the bias is a necessary premise for any such argument.

## We need to talk about science and philosophy

What can be done to facilitate and encourage debate about philosophical biases in science? Recognizing philosophical biases is a good starting point, but the responsibility for this cannot be left to the individual scientists. Instead, we need to develop a culture in the scientific community for critically discussing conceptual and meta-empirical issues: this should involve universities, research institutes and journals. Philosophers of science should contribute to this process by working to engage with students and researchers in discussions about the philosophical foundation of scientific norms, methods and practices.

At our own institute, the NMBU Centre for Applied Philosophy of Science in Norway, we find that students and researchers become interested in discussing these matters once they are made aware of them. The Norwegian higher education system has a long tradition of mandatory training in the philosophy of science for Masters and PhD students, and Polish universities are famous for the rigorous scientific training received by philosophy of science students. These initiatives point in the direction that we want to see: philosophically informed scientists and scientifically informed philosophers of science who are prepared to debate with each other on topics that are highly relevant to both.

## Note

This Feature Article is part of the Philosophy of Biology collection
